# MSC encapsulation in alginate microcapsules prolongs survival after intra-articular injection, a longitudinal in vivo cell and bead integrity tracking study

**DOI:** 10.1007/s10565-020-09532-6

**Published:** 2020-05-30

**Authors:** Sohrab Khatab, Maarten J. Leijs, Gerben van Buul, Joost Haeck, Nicole Kops, Michael Nieboer, P. Koen Bos, Jan A. N. Verhaar, Monique Bernsen, Gerjo J. V. M. van Osch

**Affiliations:** 1 Department of Orthopaedics, Erasmus MC University Medical Center Rotterdam, Wytemaweg 80, 3015 CN Rotterdam, the Netherlands; 2 Department of Radiology and Nuclear Medicine, Erasmus MC University Medical Center Rotterdam, Wytemaweg 80, 3015 CN Rotterdam, the Netherlands; 3 Department of Otorhinolaryngology, Erasmus MC University Medical Center Rotterdam, Wytemaweg 80, 3015 CN Rotterdam, the Netherlands

**Keywords:** Cell encapsulation, Cell therapy, Alginate, Mesenchymal stem cells, In vivo longitudinal imaging

## Abstract

Mesenchymal stem cells (MSC) are promising candidates for use as a biological therapeutic. Since locally injected MSC disappear within a few weeks, we hypothesize that efficacy of MSC can be enhanced by prolonging their presence. Previously, encapsulation in alginate was suggested as a suitable approach for this purpose. We found no differences between the two alginate types, alginate high in mannuronic acid (High M) and alginate high in guluronic acid (High G), regarding MSC viability, MSC immunomodulatory capability, or retention of capsule integrity after subcutaneous implantation in immune competent rats. High G proved to be more suitable for production of injectable beads. Firefly luciferase-expressing rat MSC were used to track MSC viability. Encapsulation in high G alginate prolonged the presence of metabolically active allogenic MSC in immune competent rats with monoiodoacetate-induced osteoarthritis for at least 8 weeks. Encapsulation of human MSC for local treatment by intra-articular injection did not significantly influence the effect on pain, synovial inflammation, or cartilage damage in this disease model. MSC encapsulation in alginate allows for an injectable approach which prolongs the presence of viable cells subcutaneously or in an osteoarthritic joint. Further fine tuning of alginate formulation and effective dosage for might be required in order to improve therapeutic efficacy depending on the target disease.

**Figure:**
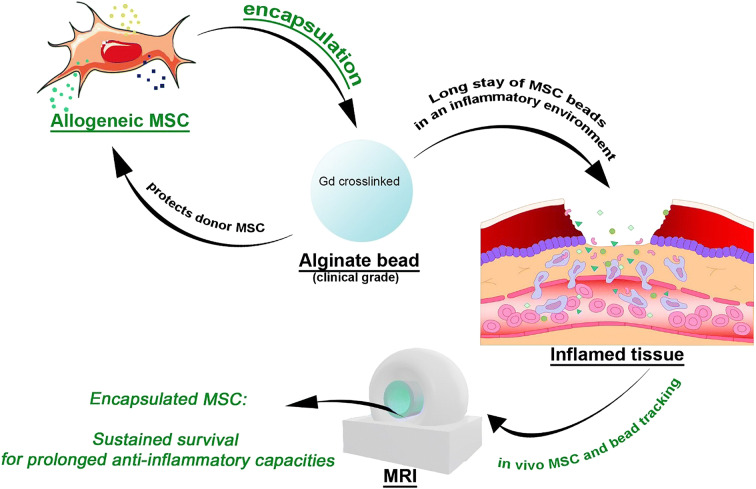
Graphical Abstract

Graphical Abstract

## Introduction

Application of mesenchymal stem cells (MSC) is promising due to their ability to influence their (micro-) environment by secreting trophic mediators (Caplan 2017; Kinnaird et al. 2004; Timmers et al. 2011; Le Blanc and Mougiakakos 2012; Prockop 2009; Ren et al. 2008). These secreted factors have been demonstrated to counteract inflammatory and catabolic processes and attract endogenous repair cells in various pathological conditions (van Buul et al. 2012; Estrada et al. 2009; Timmers et al. 2011; Prockop and Oh 2012). MSC-secreted factors have been shown to improve cardiac function after myocardial infarction in pigs (Timmers et al. 2011), improve lower limb movement after spinal cord ischemia in rats (Takahashi et al. 2018), ameliorate limb ischemia in mice (Huang et al. 2014), and reduce pain in a murine osteoarthritis (OA) model (Khatab et al. 2018b). Previously, it was demonstrated that although injection of MSC has beneficial effects, the MSC themselves are no longer detectable 3 weeks after intra-articular injection (Diekman et al. 2013; Mak et al. 2016). We hypothesize that the efficacy of MSC can be enhanced by prolonging their local presence by enabling longevity through encapsulation in a biomaterial.

Alginate is widely used in tissue engineering and drug delivery because of its biocompatibility, stability, non-antigenicity, and chelating ability (reviewed in Sun and Tan (2013); Lee and Mooney (2012)). This commonly used gel for cell encapsulation provides protection of the encapsulated cells against the host’s immune system, and at the same time retains cells at the desired location, by acting as a mechanical barrier. The increased cell retention and cell survival can result in an enhanced therapeutic efficacy at the local site of the disease (Levit et al. 2013; Serra et al. 2011). Besides providing a barrier for cells, alginate allows for the release of growth factors and cytokines produced by the encapsulated cells to the microenvironment and vice versa. Cytokines from the microenvironment can reach the encapsulated cells. This provides a setting for dynamic cross talk between cells and their environment (Sun and Tan 2013; Choi et al. 2018; Shoichet et al. 1996). Furthermore, by encapsulating cells in alginate, we may create a safer way for using allogeneic cells as an alternative to autologous grafts by shielding them from the host’s immune system (Leijs et al. 2017; Duvivier-Kali et al. 2001; de Vos et al. 2006). This would greatly enhance the clinical translatability of MSC-based therapies. We have previously shown that allogenic MSC encapsulated in alginate could survive locally after subcutaneous implantation in vivo and could act as an interactive immunomodulatory release system for at least 5 weeks in vitro, hereby emphasizing the possible advantages of this approach (Leijs et al. 2017).

The variety in composition and production methods of different alginates has a major effect on its biocompatibility, stability, non-antigenicity, and chelating ability (Lee and Mooney 2012). Therefore, the first objective of this work was to find the most suitable clinical grade alginate for MSC encapsulation to enable their longevity in vivo, while maintaining anti-inflammatory and tissue-modulating capacities. Alginate consists of a combination of β-D-mannuronic acid and α-l-guluronic acid. We compared two alginates, one consisting of a high concentration of β-d-mannuronic acid (High M alginate) and the other with high concentration of α-l-guluronic acid (High G alginate). The alginates were evaluated regarding their effect on cell survival, preservation of immunomodulatory function of the MSC, and histocompatibility using a set of in vitro assays and in vivo tests. One alginate formulation was selected to reproducibly produce small beads of injectable size*.* Then, we tested the prolonged presence of MSC and alginate microcapsules as well as their therapeutic efficacy in a local disease model.

Injection of MSC has been shown to diminish several features of osteoarthritis (OA) in pre-clinical and some initial clinical studies (Ter Huurne et al. 2012; Murphy et al. 2003; van Buul et al. 2014; Lamo-Espinosa et al. 2016; Gupta et al. 2016; Pers et al. 2016). OA is a degenerative disabling joint disease, characterized by loss of cartilage integrity, subchondral bone changes, formation of osteophytes, and inflammation of the synovial membrane (Zhang and Jordan 2010). Unfortunately, to this date, no curative treatment for OA exists, while OA is a growing problem in society, already affecting over 10% of individuals aged 60 years or older (Zhang and Jordan 2010). We evaluated whether encapsulation in alginate could prolong the local presence of allogeneic MSC in an immunocompetent rat OA model, using longitudinal bioluminescence imaging (BLI) and we followed the structural integrity of the alginate beads after injection in the knee of rats via longitudinal MRI. Since pain and functional disability are the main reasons for patients to seek medical treatment, we evaluated the efficacy of encapsulation of MSC in alginate beads to reduce pain as well as cartilage damage and synovial inflammation in a rat model of OA.

## Materials and methods

### Expansion of rat and human mesenchymal stem cells

Allogeneic rat MSC (rMSC) were used for cell tracking experiments in vivo. rMSC were isolated (with ethical approval under animal ethical no. EMC 116-12-08) from 3 to 4 months old male Lewis rats (Janvier labs) as described elsewhere and expanded up to passage 3 (Farrell et al. 2006), to be used for subcutaneous in vivo experiments. For in vivo cell tracking experiment in the joint, we used allogeneic F344 rat MSC (Millipore, Billerica, MA) that were transduced to express firefly luciferase (r(Fluc)MSCs) as described before (van Buul et al. 2014; Guenoun et al. 2013).

Human bone marrow MSC (hMSC) were used to evaluate therapeutic efficacy in vitro and in vivo. Cells were derived from 6 patients undergoing total hip replacement (mean age 49 ± 11.2 years; F:M ratio, 1:1) by needle aspiration after written informed consent and approval by the medical ethical committee (Erasmus MC protocol METC-2004-142 and Albert Schweizer Hospital protocol 2011-07). Bone marrow cells were plated at 50,000 cells/cm^2^ and after 24 h flasks were washed to remove non-adherent cells and cells were further cultured and expanded as described below for a maximum of 4 passages.

For cell expansion, both rat and human MSC were seeded at a density of 2300 cells/cm^2^ in cell culturing flasks, in expansion medium consisting of Minimal Essential Medium Alpha (αMem; Gibco, Rockville, USA), 10% heat-inactivated Fetal Calf Serum (FCS; Gibco, Rockville, USA), 1.5 μg/mL fungizone (Invitrogen, Carlsbad, USA), 50 μg/mL gentamicin (Invitrogen, Carlsbad, USA), 25 μg/mL ascorbic acid-2-phosphate (Sigma-Aldrich, Saint Louis, USA), and 1 ng/mL Fibroblast Growth Factor 2 (FGF2; AbD Serotec, Oxford, UK). Cells were cultured in an incubator at 37 °C, 5% CO_2_, and 90% humidity. Medium was renewed twice a week. When MSCs were approximately 70% confluent, they were passaged by trypsinization of cells with a 0.25% trypsin/EDTA solution (Life Technologies, Waltham, USA).

### Preparation of MSC-alginate constructs

Clinical-grade high mannuronate (M) alginate (*Laminaria pallida*) and high guluronate (G) alginate (*Laminaria hyperborea*) (respectively; Lot no. E01 AAL-070912 and Lot no. C01 AAL-110808 both kind gifts of BTG/CellMed AG, Alzenau, Germany) were used. Both alginates were diluted in a 0.5%, 1.1%, and 2.5% concentration in NaCl 0.9% and filter-sterilized afterwards. The shear-dependent viscosity of the solutions was measured by a rheometer Physica MCR301 (Anton Paar GmbH, Ostfildern, Germany) at room temperature (20 °C). The viscosity was measured in a shear rate range of 1–5000 s^-1^ by increasing the shear rate every 5 s for a duration of 2 min and 45 s. Data were analyzed with Rheoplus Software version 3.4 (Anton Paar GmbH, Ostfildern, Germany). For 1.1% High M alginate, the low shear viscosity at 20 °C was found to be 1320 mPa s; for 1.1% High G alginate, the low shear viscosity at 20 °C was 274 mPa s. The effect of shear stress on the viscosity was similar for both alginates.

Prior to encapsulation, MSC were washed with saline. A homogeneous solution of 4.0 × 10^6^ MSC per 1 mL filter-sterilized 1.1% High M alginate or 1.1% High G alginate was prepared. This cell density was selected after a series of tests comparing 0.4, 4, and 20 million cells/mL, indicating that 4 million cells/mL was the most efficient cell number in terms of cell viability and immunomodulatory properties during 2 weeks encapsulation in alginate in vitro (data not shown).

Beads of approximately 2 mm in diameter were created by manually dripping the MSC-alginate mixture through a 23-gauge needle in 102 mM CaCl_2_ solution for 10 min. After incubation, beads were washed two times for 5 min with saline before further use in in vitro experiments.

For subcutaneous implantation, alginate disks were created by polymerization of the rMSC-alginate solution which took place in a sterilized, custom-designed mold consisting of two durapore membranes (5-μm pore size, Millipore) at both sides of a 3-mm-thick metal ring (Wong et al. 2001). After 30 min in 102 mM CaCl_2_, the construct was washed two times in saline and 8-mm-diameter constructs were made with sterile dermal punches (Spengler, Hanover, Germany).

To produce smaller beads in a more reproducible way, we used the Buchi Encapsulator B-395 Pro (Buchi Labortechnik AG, Flawil, Switzerland). After optimizing the settings, beads of approximately 300 μm in diameter were made from 1.1% High G alginate with the following machine settings: flow rate 3 mL/min, nozzle size 150 μm, frequency 1600 Hz, voltage 730 V, stir-rate 30% speed. To be able to track the alginate beads using MRI in vivo, we solidified the alginate solution with 102 mM CaCl_2_ with 20 mM gadolinium (III) chloride hexahydrate (Lot no. MKBJ3153V, Sigma-Aldrich, St. Louis, USA). Beads were kept in this solution for 10 min, then washed twice with saline solution, and kept for a maximum of 4 h in saline prior to injection.

### In vitro characterization of MSC-alginate constructs

Three hMSC-alginate beads were placed in 24-well plates in 900 μL of medium consisting of αMem with fungizone (1.5 μg/mL), gentamicin (50 μg/mL), 1% insulin-transferrin-selenium (ITS; Biosciences, New Jersey, USA), and 0.1 mM vitamin C (Sigma, St. Louis, MO). Medium was refreshed twice a week. Beads were harvested directly after encapsulation and washing with saline (*T* = 0), after 1 week (*T* = 1), and 2 weeks of culture (*T* = 2) to determine cell viability and immunomodulatory capacity.

#### Cell viability

Survival of encapsulated hMSC was measured by the amount of DNA and LIVE/DEAD® assay at *T* = 0 and *T* = 2 weeks (using cells from 2 different bone marrow donors). For DNA analyses, six beads were harvested at each time point and dissolved in 150 μL/bead. Sodium-citrate buffer (150 mM NaCl (Sigma-Aldrich, St. Louis, USA), 55 mM Na-citrate (Sigma-Aldrich), 20 mM EDTA (Sigma-Aldrich)) for half an hour at 4 °C. Samples were centrifuged at 180×*g* for 8 min and pellets were stored at − 80 °C. Standard curves were made with DNA of hMSC of the same donor before encapsulation. DNA was determined with the CyQUANT® Cell Proliferation Assay Kit (Invitrogen, Carlsbad, CA, USA) following the manufacturer’s instructions. The fluorescence measurements were performed on a microplate reader with excitation at 480 nm and emission detection at 520 nm (Spectramax Gemini, Molecular Devices, Sunnyvale, CA, USA).

LIVE/DEAD® assay (Invitrogen, Carlsbad, CA, USA) was performed by incubating MSC-alginate beads for 30 min in 100 μl labelling solution with 1.0 μL/mL green-fluorescent calcein-AM and 1.5 μL/mL red fluorescent ethidium homodimer-1, at 37 °C. Z-stacks were made using an Axiovert 200 MOT fluorescent microscope (Carl Zeiss microscopy, Thornwood, NY, USA) with a thickness of 200 μm per slide. Viable and dead cells were counted in two Z-stacks on two areas of 0.25 mm^2^ per Z-stack using ImageJ 1.48 (Java, Redwood Shores, CA, USA).

#### Immunomodulatory capacity

First, immunomodulatory capacity of the encapsulated hMSC (using cells from 2 different bone marrow donors) was determined by measuring interleukin-6 (IL-6) protein levels and IDO activity. After 2 weeks of culture, hMSC were stimulated with 50 ng/mL IFNγ and 50 ng/mL TNFα (Peprotech, London, UK). For control, medium without IFNγ and TNFα was added to encapsulated hMSC. After 24 h, conditioned medium was harvested and stored at – 80 °C until analyses. IL-6 levels in the stimulated and non-stimulated hMSC conditioned media were measured by ELISA (R&D systems, Abingdon, UK) according to the manufacturer’s instructions. IDO activity was determined in the stimulated and non-stimulated MSC conditioned media by the level of its metabolite l-kynurenine. This was measured spectrophotometrically as described previously (Kang et al. 2008).

The immunosuppressive capacity of encapsulated hMSC was determined in a co-culture with activated lymphocytes. The MSC-alginate beads (using MSC from 1 bone marrow donor) were cultured for 2 days and 29 days and then were stimulated with 50 ng/mL IFNγ and 50 ng/mL TNFα for 24 h. The MSC-alginate beads were washed two times with saline and 4, 2, or 1 bead (approx. 3.0 × 10^4^ hMSC per bead) was transferred in a 48-well plate to obtain a 1:2.5, 1:5, and 1:10 MSC/peripheral blood mononuclear cells (PBMCs) ratio. PBMCs were isolated with Ficoll-Paque™ PLUS (density 1.077 g/mL; GE Healthcare, Uppsala, Sweden) from buffy coats of healthy blood donors (Sanquin, Rotterdam, The Netherlands) and frozen at − 150 °C until further use. In total, 1.0 × 10^6^ PBMCs/mL were labelled with 1 μM carboxyfluorescein succinimidyl ester (CFSE) and activated with antibodies against CD3 and CD28 (1 μL per 1 × 10^6^ cells in 1 mL, BD Biosciences). As positive and negative lymphocyte proliferation control, activated and non-activated CFSE-PBMCs were used. As a positive control for immunomodulatory capacity of hMSC, 1.2 × 10^5^ hMSC in monolayer were used. After 5 days of co-culture, PBMCs were retrieved, and incubated with CD4 (APC-A; BD Biosciences) and CD8 (PE-CY7-A; BD Biosciences). Proliferation was determined from dilution of CFSE (FITC) staining using 8 colors FACSCANTO-II with FACSDIVA Software (BD Biosciences) and FlowJo Software (Tree Star Inc., Palo Alto, CA).

### Animal experiments

**Fig. 1:**
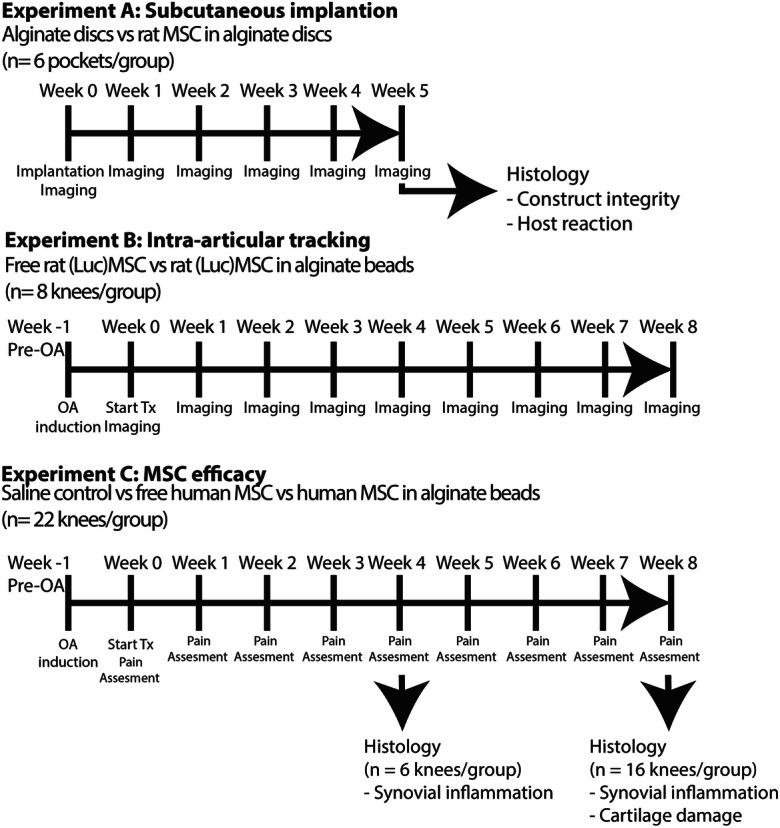
Experimental set up of in vivo experiments to evaluate the effect of encapsulation of MSC in alginate on cell viability and efficacy to treat OA. In experiment A, allogeneic rMSC-alginate constructs and empty alginate constructs were implanted subcutaneously in rats to assess construct integrity and MSC survival in vivo*.* In experiment B, longevity of MSC in an OA knee joint was tested using allogenic r(Fluc)MSC either free or encapsulated in alginate beads. Weekly imaging with MRI for construct integrity and BLI for cell viability followed until the end of the experiment at week 8. In experiment C, the therapeutic efficacy of hMSC in an OA knee joint was studied. hMSC were injected intra-articularly either free or encapsulated in alginate beads and compared with saline control. The effect on pain was measured weekly and knees were harvested for histology at week 4 (synovial inflammation) and week 8 (synovial inflammation and cartilage damage)

We performed three separate animal experiments to assess influence of MSC encapsulation on cell longevity and effect of encapsulation on treatment efficacy. These experiments were carried out in accordance with the EU Directive 2010/63/EU for animal experiments. First, we implanted rMSC-alginate (High G and High M) constructs subcutaneously in rats to asses construct integrity and rMSC survival in vivo (experiment A, Fig. 1). In the second in vivo experiment, we moved to the joint and traced intra-articularly injected r(Fluc)MSC and r(Fluc)MSC-alginate High G beads cross-linked in the presence of gadolinium, over time to prove that we can prolong the presence of rMSC at the desired location (experiment B, Fig. 1). In the third experiment, we studied the therapeutic efficacy of intra-articularly injected hMSC either free or encapsulated in beads (experiment C, Fig. 1). All experiments are explained in further detail below. All experiments were performed on 16-week-old male Wistar rats, weighing 250–300 g (Harlan Netherlands BV, The Netherlands), with approval of the animal ethics committee (protocol no. EMC116-15-02). Rats were housed in groups of two per cage, under 12-h light-dark cycle at a temperature of 24 °C, and had access to water and food ad libitum at the animal testing facilities of the Erasmus MC, University Medical Center. Before the start of the experiments, rats were allowed to acclimatize for a week. All procedures involving subcutaneous implantations, intra-articular injections, or scanning were applied under 2.5% isoflurane anesthesia.

#### Subcutaneous implantation

The constructs of High G alginate and High M alginate with rMSC were placed in saline and subcutaneously implanted on the back of three rats. Each rat received two constructs of High G alginate with rMSC and one without cells and two constructs of High M alginate with rMSC and one without cells. Directly and 12 h after the operation, the rat got a subcutaneous injection with buprenorphine (Temgesic) 0.01 mg/kg bodyweight. To track the subcutaneously implanted rMSC, they were labelled 1 day prior to encapsulation in alginate with superparamagnetic iron oxide (SPIO) using ferumoxides 100 μg/mL medium (Endorem™, Guerbet S.A., Paris, France) complexed to protamine sulphate 5 μg/mL medium (LEO Pharma N.V., Wilrijk, Belgium) as described previously (van Buul et al. 2009). Imaging of the MSC constructs was done by MR imaging directly after implantation and thereafter weekly up to 5 weeks. Five weeks after implantation, the rats were euthanized. The subcutaneous implantation regions were harvested, fixed in 0.05 M Tris-buffered saline with 10 % formalin and 15 mM CaCl for 24 h and embedded in paraffin.

#### Intra-articular cell tracking experiments

To evaluate bead integrity and the retention of encapsulated cells in a diseased environment that is mechanically loaded, we induced knee OA in rats. Mono-iodoacetate (MIA; 300 μg) was intra-articularly injected bi-laterally in 25 μL of saline (van Buul et al. 2014) using a 50-μL glass syringe (Hamilton Company, Ghiroda, Romania) and a 27-G needle (Becton, Dickinson and Company, Benelux N.V. Belgium). One week after OA induction (referred to as day 0), rats were randomly divided into two treatment groups: (1) freely injected 1.0 × 10^5^ r(Fluc)MSC (*n* = 8 knees), and (2) approx. 1.0 × 10^5^ r(Fluc)MSC encapsulated in gadolinium-labelled High G alginate beads (*n* = 8). Beads and loose cells were both injected in a total volume of 25 μL saline. These injections were done with a 250-μL glass syringe and a custom made 23-G needle (Hamilton Company, Ghiroda, Romania). The choice of this relatively low cell number was based on the assumption that with longer presence of cells the number of cells needed for a therapeutic effect would be lower. In a previous study, we found an analgesic effect of 1.0 × 10^6^ freely injected cells in the same OA model (van Buul et al. 2014).

To follow up cell viability and alginate bead integrity, weekly bioluminescence and MR imaging were performed (methods see below). Animals were scanned once directly after injection of the cells and hereafter once a week for a total of 8 weeks. Animals were euthanized 8 weeks after treatment.

#### Intra-articular hMSC efficacy experiment

Bilateral OA was induced as described above. One week after OA induction (referred to as day 0), rats were randomly divided into three treatment groups, and rats received in both knees the same treatment, except one animal which received free hMSC in one knee and saline control in the contra-lateral knee resulting in three groups: (A) saline control (*n* = 19); (B) 1.0 × 10^5^ freely injected hMSC (*n* = 19); (C) 0.8 × 10^5^ ± 0.1 × 10^5^ hMSC encapsulated in alginate beads (*n* = 22). MSC from 3 human donors were pooled to take into account the inter-donor variability. Four weeks after treatment, the animals were euthanized to assess the effects of our treatments on synovial inflammation and knee joints were prepared for histological evaluation (*n* = 6 knees/group). The remaining animals were euthanized 8 weeks after start of treatment and knee joints were harvested for histological analysis (*n* = 16 knees/group). In the latter group, pain was evaluated weekly with mechanical allodynia tests (method see below).

### Imaging

#### Bioluminescence imaging (BLI)

To evaluate the presence of living cells over time, luciferase activity of injected r(Fluc)MSC was measured using the Xenogen IVIS Spectrum (PerkinElmer, Hopkington, MA), 15 min after intra-peritoneal injection of 50 μg beetle luciferin in 150 μL saline (Promega Benelux B.V., Leiden, the Netherlands). Optical intensity is reported as arbitrary units. Data were analyzed using the software Living Image version 3.2 (Caliper LS).

#### Magnetic resonance imaging (MRI)

MR imaging was performed on a preclinical 7.0-T MRI scanner (MR 901 Discovery, Agilent/GE Healthcare, Milwaukee, WI). For imaging SPIO-labelled rMSC, a 72-mm transmit/receive body coil was used. Image acquisition was performed using a fast spoiled gradient echo sequence with the following parameter settings: TE/TR = 1.1/7.3 ms, NEX = 4, FOV 8 × 6 cm^2^, acquisition matrix 256 × 192, slice thickness = 1 mm, bandwidth = 60 kHz, 16 degrees. Sagittal and coronal scans were performed to localize the hypo-intense SPIO deposits.

For intra-articular localization of alginate beads and to follow up the presence of these beads in vivo, we used gadolinium in the alginate beads and scanned with a 150-mm body coil for transmission, and a four-channel cardiac coil (Rapid BiomedGmbH, Rimpar, Germany) for signal reception. A 3D, fast spoiled gradient echo sequence was used to scan the injected rat knees (TE/TR 10.0/30.0 ms, NEX 2, FOV 6.00 × 4.50 cm^2^, acquisition matrix 512 × 512, slice thickness 0.50 mm, bandwidth 31.25 kHz, flip angle 16°). The number of beads per knee was counted manually using the built-in DICOM viewer on the scanner (Software build 1094.1, General Electric Healthcare, Milwaukee, WI).

### Pain assessment

Hind paw withdrawal reflex was measured with von Frey filaments (Bioseb, France) as an indicator of pain (Koda et al. 2014). Animals were habituated to measuring cages and handling by the examiner starting 2 weeks prior to OA induction. The hind paws of the rats were stimulated using a series of von Frey filaments, increasing in strength starting at 0.2 to a maximum of 26 g. If the paw was withdrawn after the administration of the von Frey filament for a minimum of 4/5 times, the strength of the filament was noted. If no reaction was seen after 5 attempts, for a maximum of 3 s each, a stronger filament was used until a response was measured. A baseline measurement was performed after the rats were habituated and just before OA induction. Follow-up measurements were performed 7 days after OA induction, which was just before therapy administration, and thereafter once weekly until the end of the experiment at 8 weeks. All measurements were performed by the same examiner, blinded for the treatment groups, in the same room, with temperature set at 18–20 °C and the same background noises present at time of measurement. Measurements were performed at the same time of day.

### Histology

#### Evaluation of subcutaneously implanted MSC-alginate constructs

Paraffin sections (6 μm) were deparaffinised and stained for Perls’ iron according to the manufacturer’s protocol (Klinipath BVBA, Duiven, The Netherlands) to locate the SPIO-rMSC. SPIO-labelled rMSC are stained blue with Perls’. CD68 and CD3 staining was performed to identify macrophages and T lymphocytes as an indication of a local inflammatory response. Antigen retrieval for CD68 and CD3 was performed through incubation in citrate buffer (10 mM citric acid, 0.05% Tween20, pH 6.0) for 20 min at 90–95 °C. Sections were incubated for 1 h with primary antibodies for CD68 (BM4000 5 μg/mL; OriGene Technologies, Herford) or CD3 (Ab16669, dilution 1:100; Abcam Cambridge, UK) diluted in PBS/1 %BSA (Sigma no. A7284) after blocking of non-specific binding sites with 10 % goat serum (Southern Biotech no. 0060-01) in PBS/1%BSA. A secondary antibody biotinylated goat anti-mouse 1:50 (Biogenex, HK-325-UM) was used, followed by incubation with streptavidin-AP 1:50 (Biogenex, HK-321-UK). Staining was then visualized using an alkaline-phosphatase substrate followed by counterstaining with hematoxylin.

#### Evaluation of knee joints after MSC-alginate bead injection

Knees were fixed in formalin 4% (v/v) for 1 week, decalcified in 10% EDTA for 2 weeks, and embedded in paraffin, and coronal sections of 6 μm were cut. Sections were collected anterior to posterior every 300 μm to give a good overview of the damage throughout the entire knee. Cartilage damage was evaluated on Safranin O-stained sections, with a scoring system described by Pritzker et al. (2006). Scoring was done on three sections aiming around the mid-portion of the joint. The Pritzker score ranges from 0–6 for structural damage and 0–4 for GAG staining intensity. These scores were multiplied with a factor 1–4 to account for the percentage of surface affected (factor 1, 0–25%; 2, 26–50%; 3, 51–75%; 4, 76–100% surface area). This led to a maximum score of 24 for structural damage and a maximum of 16 for GAG loss, as described previously by van Buul et al. (2014) The scoring of two blinded observers was averaged and used for data analyses.

Synovial inflammation was evaluated on sections stained with hematoxylin–eosin. The sections were imaged using NanoZoomer Digital Pathology program (Hamamatsu Photonics, Herrsching am Ammersee, Germany), and synovial thickness was measured from the capsule to the superficial layer of the synovial membrane in the parapatellar recesses at the medial and the lateral sides at three positions per section, as previously described Khatab et al. 2018a; Khatab et al. 2018b). These measurements were performed on three sections per knee, with 300 μm between the sections. The thickness measurements were averaged to obtain a single value per knee joint.

### Statistical analysis

Data was analyzed with IBM SPSS statistics 24 (SPSS, Chicago, IL). To evaluate the in vitro data of DNA, live/dead cell count, IL-6 secretion, IDO activity, and lymphocyte proliferation of MSC-alginate beads, Mann-Whitney *U* tests were performed. To evaluate the number of alginate beads on MRI scans of rat joints over time, a Wilcoxon signed-rank test was performed, since data did not meet requirement for normality with the Shapiro-Wilk test. To compare fluorescence intensity of r(Flu)MSC in the free MSC group vs. the MSC-alginate group, a Mann-Whitney *U* test was performed, since data did not meet the requirement of equal distribution and normality with the Shapiro-Wilk test. To evaluate the fluorescence intensity within groups over time, a Wilcoxon signed-rank test was performed. For treatment effects on pain, all groups were compared using a linear mixed model in which measurement time point and treatment were considered fixed factors and withdrawal threshold a dependent factor. After significance was confirmed, a one-way ANOVA was performed to determine differences between groups. To determine differences over time per treatment, a linear mixed model analysis was performed in which measurement time point was considered a fixed and withdrawal threshold a dependent factor. Post hoc analysis using Bonferroni correction was performed.

For synovial inflammation, homogeneity of variances and normality were confirmed with a Shapiro-Wilk test. Next an one-way ANOVA was performed; post hoc analyses were performed and Bonferroni correction was applied.

For non-parametric cartilage scoring data, Mann-Whitney *U* tests were used to assess MIA or measurement time point effects. Kruskal Wallis tests were used for treatment effects within time points. Post hoc analyses were performed by Bonferroni correction. For all tests, *p* values < 0.05 were considered statistically significant.

## Results

### MSC remain viable and immunomodulatory active in both clinical grade High M alginate and High G alginate

**Fig. 2:**
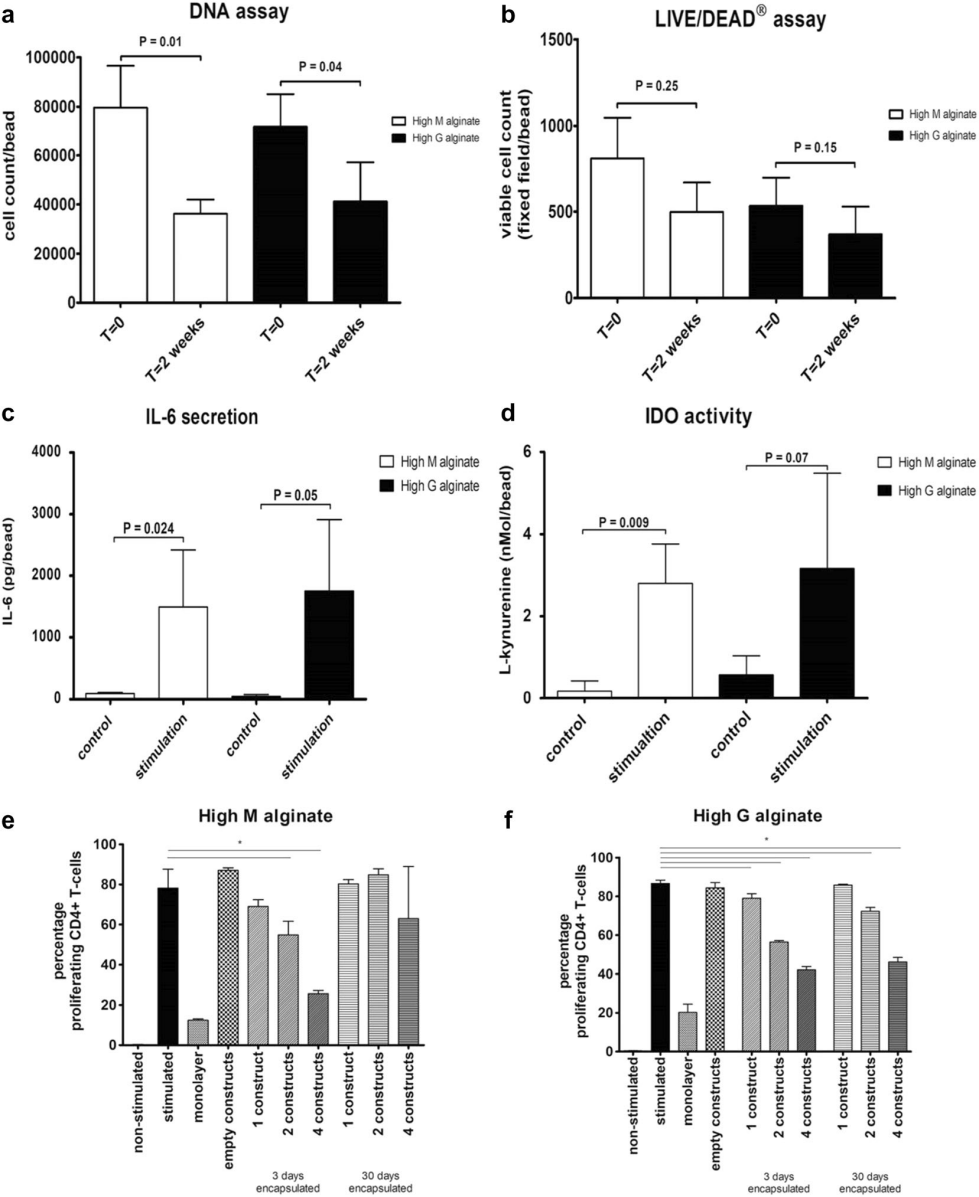
Viability and immunomodulatory capacity of encapsulated MSC in High G alginate and High M alginate. **a** DNA amount directly after encapsulation or after 2 weeks. **b** Number of viable cells directly after encapsulation and after 2 weeks. **c** IL-6 secretion and **d** IDO activity measured as concentration of l-kynurenine in the medium after stimulation with IFNγ/TNFα (**a**–**d** all performed with hMSC of 2 different donors with 3 samples per donor). Activated CD4+/CD8+ T lymphocytes co-cultured with one, two, and four hMSC-alginate constructs with **e** High M or **f** High G alginate, 3 days and 30 days after encapsulation of hMSC (performed in triplicate with samples of 1 hMSC donor and 1 PBMC donor). First bar: non-stimulated PBMCs; positive control. Second bar: stimulated PBMC without alginate constructs. Third bar: stimulated PBMC in the presence of 1.2 × 10^5^ hMSC in monolayer. Fourth bar: stimulated PBMC in the presence of empty alginate constructs. Mean ± SD is shown * indicates statistical significance

The amount of DNA measured in the beads after 2 weeks was 45.4% in High M alginate (*p* = 0.01) and 57.4% in High G alginate (*p* = 0.04) of the amount at the moment of encapsulation (Fig. 2a). No significant difference was found in the amount of DNA between High M alginate and high G alginate constructs. The number of viable cells was not significantly different between High M and High G alginates directly after encapsulation or after 2 weeks in culture (Fig. 2b).

hMSC encapsulated in either alginate retained their immunomodulatory capacities when stimulated with IFNγ and TNFα. This stimulation induced IL-6 secretion (Fig. 2c) and IDO activity (Fig. 1d) from the encapsulated MSC irrespective of the type of alginate used. Alginate-encapsulated hMSC significantly inhibited proliferation of stimulated CD4+ and CD8+ T lymphocytes. Three days after encapsulation hMSC encapsulated in High G and High M alginates (Fig. 2e, f) significantly inhibit T lymphocyte proliferation in a dose-dependent manner (all *p* = 0.024). Thirty days after encapsulation, inhibition was reduced but in particular still present in High G alginate when four and two beads were used (*p* = 0.024) (Fig. 2f). Empty constructs of alginate had no effect on T cell proliferation. The inhibition by 1.2 × 10^5^ hMSC in monolayer was similar to the inhibition of 4 alginate constructs, containing a similar number of MSC on day 0.

### No difference in construct integrity and MSC retention after in vivo implantation of encapsulated allogeneic MSC in High M alginate and High G alginate

**Fig. 3:**
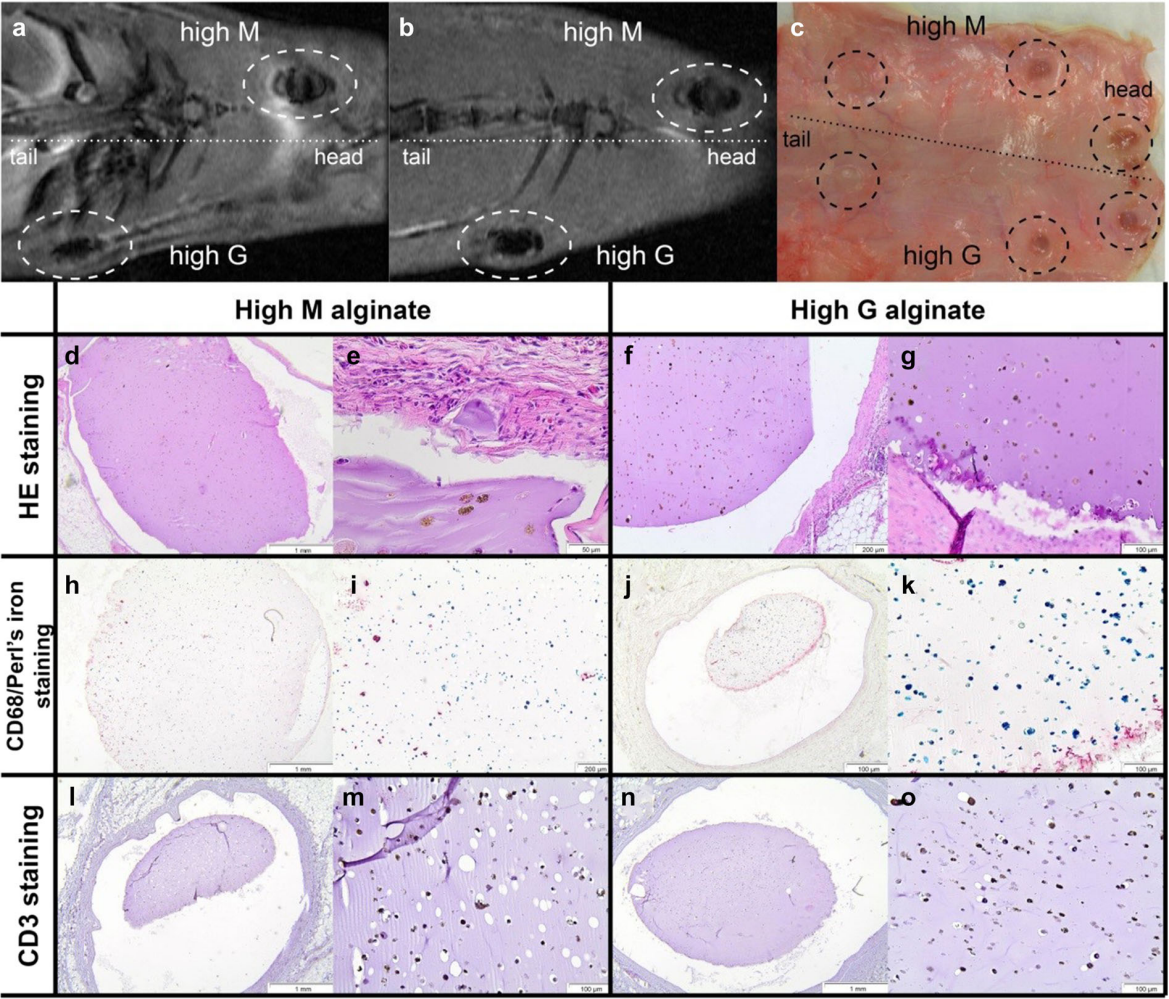
Subcutaneous implanted allogeneic rMSC in immunocompetent rats. **a** MRI image directly after implantation and **b** MRI image of the same animal 5 weeks after implantation. Alginate constructs are visible due to the labelled SPIO cells in the constructs. **c** After 5 weeks, the constructs were clearly visible after removal of the skin post-mortem. **d–g** Hematoxylin and eosin-stained tissue sections of biopsies taken at the site of the SPIO-labelled-MSC-containing construct implants of high M and high G alginate constructs. **h–k** Staining of corresponding sections shown in **d–g** with Perl’s iron staining (blue), which stains SPIO combined with CD68 staining (red) to stain macrophages. **l–o** CD3 staining (red) to stain T lymphocytes (black dots in cells represent SPIO particles)

Subcutaneously implanted alginate-encapsulated SPIO-MSC remained clearly visible on MR images over 5 weeks (Fig. 3a, b) and where clearly visible macroscopically upon explantation (Fig. 3c) without noticeable differences between high M and high G alginate constructs. As observed in histological sections, there was good integrity of the constructs (Fig. 3d–g) and a homogenous distribution of SPIO-labelled cells in alginate constructs (Fig. 3h–k). The rat tissue surrounding the constructs showed very limited foreign body reaction without cell infiltration of macrophages (CD68; Fig. 3h–k) or T lymphocytes (CD3; Fig. 3l–o). No differences in construct morphology or foreign body reaction were observed between High M alginate and High G alginate.

### Alginate encapsulation using a micro-encapsulator results in small injectable beads with vital MSC.

**Fig. 4:**
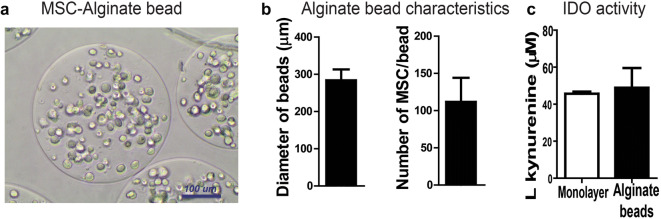
Characteristics of MSC-alginate beads produced with micro-encapsulator device. **a** hMSC-alginate beads prepared with the micro-encapsulator. **b** The average diameter of the alginate beads and number of hMSC/bead. **C** Concentration of L-kynurenine as measure of IDO activity corrected for the number of cells in the secretome of MSC stimulated with TNFα/IFNү

After optimizing the settings of the encapsulator device, we were able to produce homogenous beads of 0.3 mm using High G alginate. With the more viscous High M alginate, the beads were larger and the size was less homogenous. We decided to continue with High G alginate. The average bead size produced with High G alginate was 284 ± 28 μm, with each bead containing 112 ± 32 MSC (Fig. 4a, b). To confirm that the anti-inflammatory capacity of the hMSC was not affected by the procedure with the micro-encapsulator, we performed an IDO assay on the secretome of the stimulated hMSC. We compared hMSC in monolayer vs. hMSC-encapsulated in alginate beads (*n* = 3 donors). Encapsulated hMSC displayed similar IDO activity compared with hMSC in monolayer (l-kynurenine concentration; 48.91 ± 10.67 μM vs. 45.63 ± 1.17 μM respectively using equivalent numbers of cells) (Fig. 4c). This indicates that after cell encapsulation, hMSC maintained anti-inflammatory capacities.

### Intra-articularly injected MSC-alginate beads remain present and metabolically active in the joint for at least 8 weeks in vivo

Unfortunately, one rat of the group with alginate died during imaging at day 0 probably due to anesthesia-related issue and the results of these knees were excluded from analyses. The other animals were longitudinally followed by imaging in MRI and BLI during 8 weeks.

**Fig. 5:**
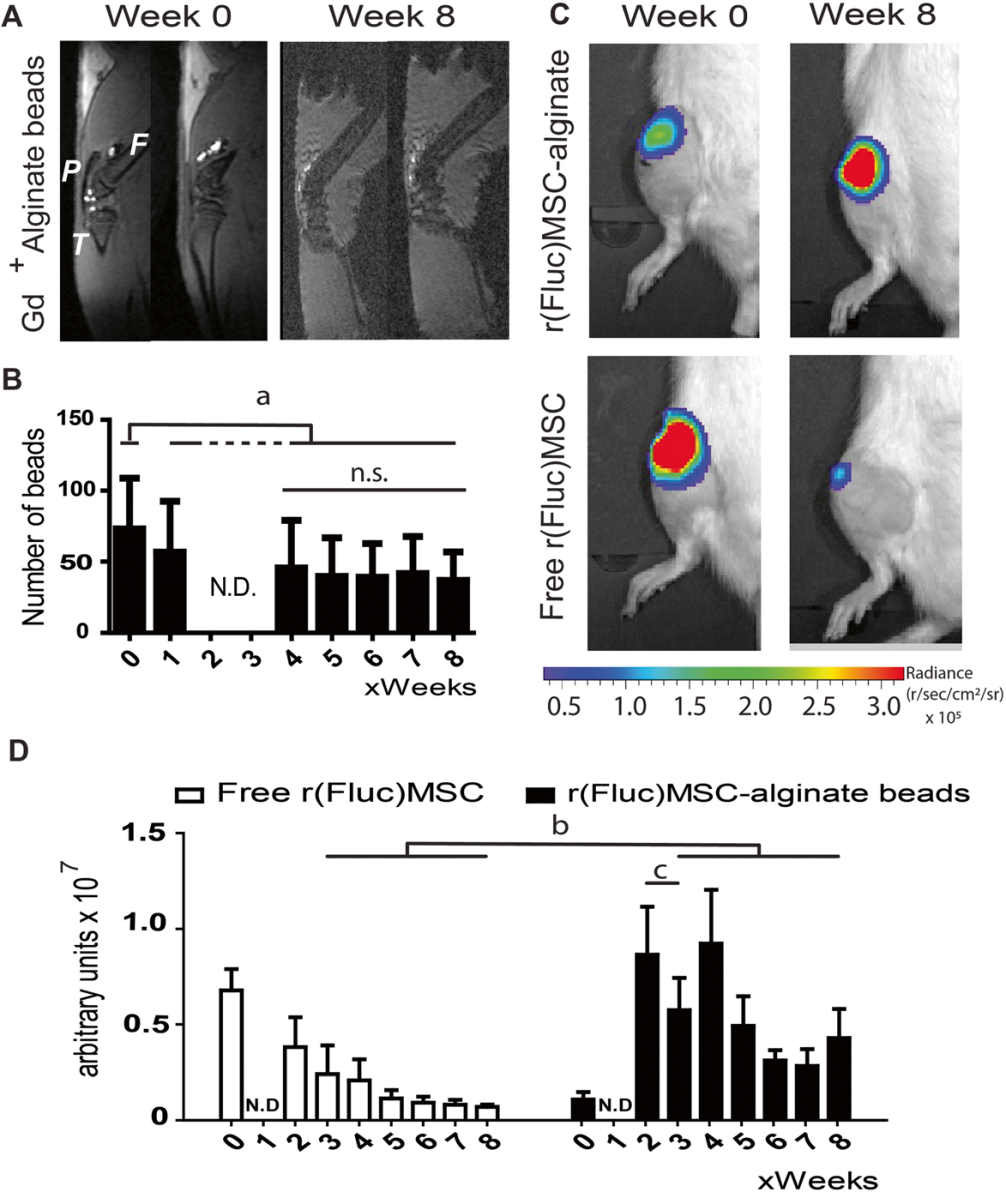
In vivo cell tracking. **a** MRI of rat knee joints injected with gadolinium-labelled alginate beads, directly after intra-articular injection and after 8 weeks. **b** Quantification of the number of alginate beads per joint over time. (Due to technical problems with the MRI scanner, week 2 and 3 scans were not available). **c** BLI of free r(Fluc)MSC and r(Fluc)MSC-alginate bead directly after injections and after 8 weeks. **d** Quantification of BLI signal over time (due to technical problems with the IVIS, week 1 scans were not available). The images shown in **a** and **c** are representative animals for each group. In **a**: P = patella, F= femur, T = tibia. In **d**: white bars = free (Fluc)MSC and black bars = r(Fluc)MSC-alginate beads. (**b**, *p* < 0.04; **c**, *p* = 0.028). *n* = 8 knees for free r(Fluc)MSC and *n* = 6 knees for r(Fluc)MSC-alginate group. N.D. = not determined due to technical error. n.s. = not significant

To track the MSC-alginate beads in vivo, alginate was cross-linked with gadolinium ions which are visible on MRI. At baseline, the number of alginate beads per knee was 73 ± 36 (Fig. 5a, b). A majority of the alginate beads were located in the suprapatellar pouch. On follow-up scans, the alginate beads appeared more dispersed throughout the joint. The number of beads decreased to 46 ± 34 per knee at week 4 (*p* = 0.028 compared with week 0), and remained stable afterwards until the end of the experiment at week 8 (37 ± 20). To track long-term cell activity after intra-articular injection, we used bioluminescence (BLI) scanning of allogeneic r(Fluc)MSC that were either encapsulated in alginate beads before injection (*n* = 6 knees) or freely injected in the knee (*n* = 8). The first scan was preformed immediately after injection (Fig. 5c) and subsequently scanned repeatedly until week 8. The BLI signal in the r(Fluc)MSC-alginate group was lower than expected based on cell number at day 0, most likely due to impaired metabolic activity of the cells shortly after encapsulation in alginate which is supported by a higher BLI signal after 2 weeks. BLI signal decreased significantly from week 2 to week 3 (*p* = 0.028) but remained stable hereafter (*p* > 0.293). From week 3 on, the fluorescence in the r(Fluc)MSC-alginate group was significantly stronger than that in the free r(Fluc)MSC group (*p* < 0.04 for all time points (Fig. 5c, d).

### Encapsulation in alginate did not improve effect of hMSC on pain, cartilage damage, or synovial inflammation

**Fig. 6:**
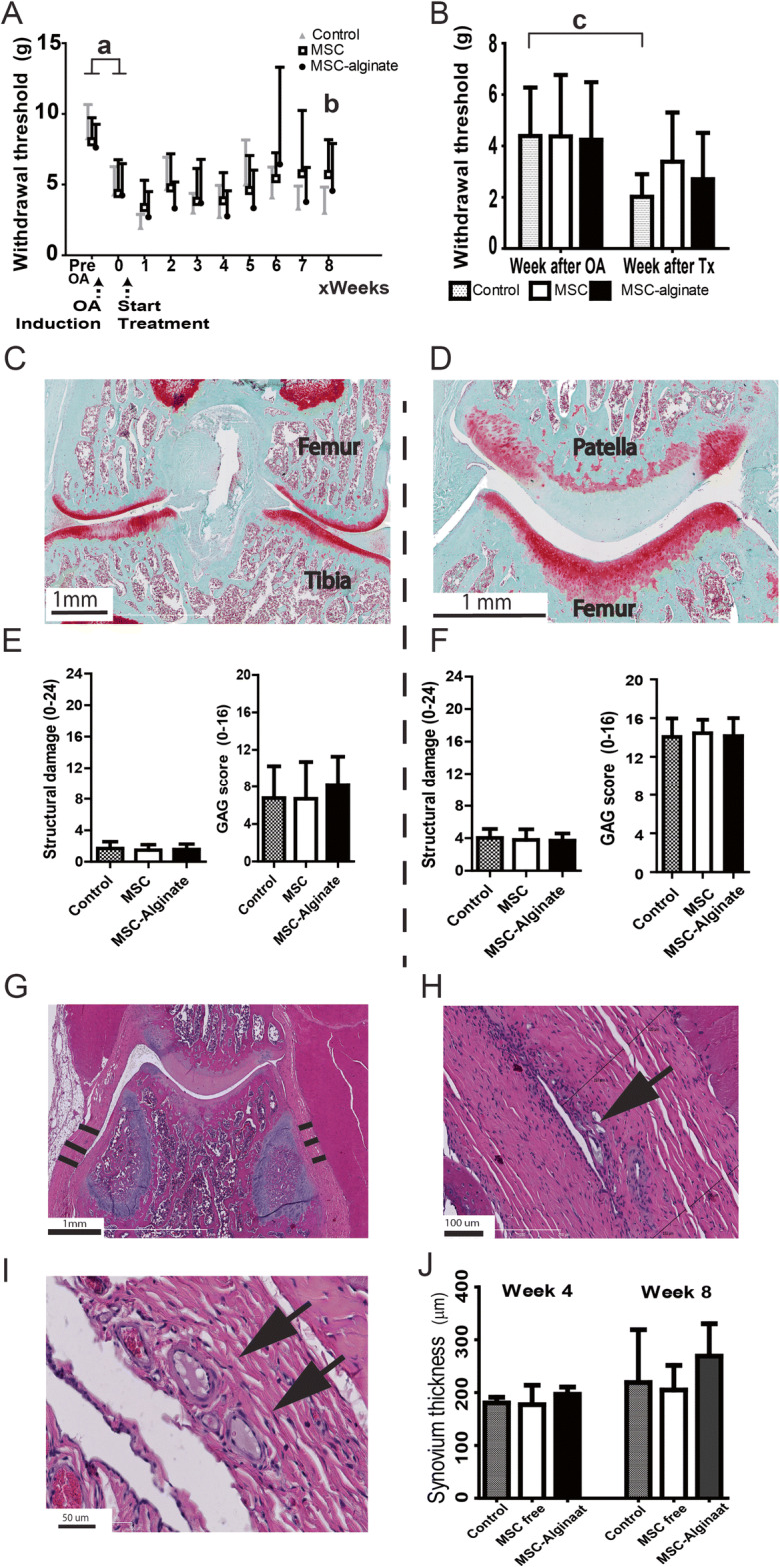
Therapeutic efficacy of MSC-alginate beads in a rat OA model. **a H**ind limb withdrawal threshold as measure of pain over time. **b** Withdrawal threshold 1 week after treatment. **c** A representative example of the Safarin-O staining at the femorotibial compartment and **d** at the patellofemoral compartment. **e** The structural damage according to the Prizker score and GAG loss for femorotibial; **f** structural damage and GAG loss in patella. The maximum score for structural damage was 24 and for GAG loss 16, in which a higher score represents more damage. **g** HE staining of parapatellar recesses and indication of synovial membrane thickness. **h–i** Some degradation and encapsulation of alginate was observed (black arrows). **j** Quantification of synovial thickness over time (**a** *p* < 0.02, **b** *p* = 0.036, **c** *p* = 0.001). All data shown as mean ± SD. At week 4, *n* = 5 knees/group; week 8, *n* = 16 knees/group

To test the efficacy of the hMSC-alginate beads as therapy for osteoarthritis, we assessed the effect on pain reduction, cartilage damage, and synovial inflammation in a rat OA model. Pain was assessed by means of tactile allodynia using the von Frey filaments. Prior to MIA injection, all the animals had comparable withdrawal thresholds. One week after MIA injection and before treatment, all three treatment groups (saline control, free hMSC, and hMSC-alginate beads) showed a significant decrease in withdrawal threshold (*p* < 0.02), indicating pain as a result of MIA injection. One week after treatment, only the animals in the saline control group showed an additional significant decrease in withdrawal threshold compared with the time point just before treatment (*p* = 0.001), indicating exacerbating pain over time. No increase in sensitivity to pain stimulus was observed in the free hMSC or hMSC-alginate beads group. Although rats in the free hMSC group showed a trend toward less pain in time, a significant difference compared with the saline-treated group was only reached at the end of the experiment at week 8 (saline control vs. free hMSC, *p* = 0.036). The hMSC-alginate beads group was not significantly different from saline control or free hMSC at week 8 (resp. *p* = 0.404 and *p* = 0.722), or any other week (Fig. 6a, b).

Cartilage damage was scored 8 weeks after treatment on the femorotibial compartment of the joint as well as the patella using a modified Pritzker score method. Mild osteoarthritic changes were present in all groups. There were no significant differences in cartilage damage or GAG loss between treatment groups (Fig. 6c–f).

**Fig. 7:**
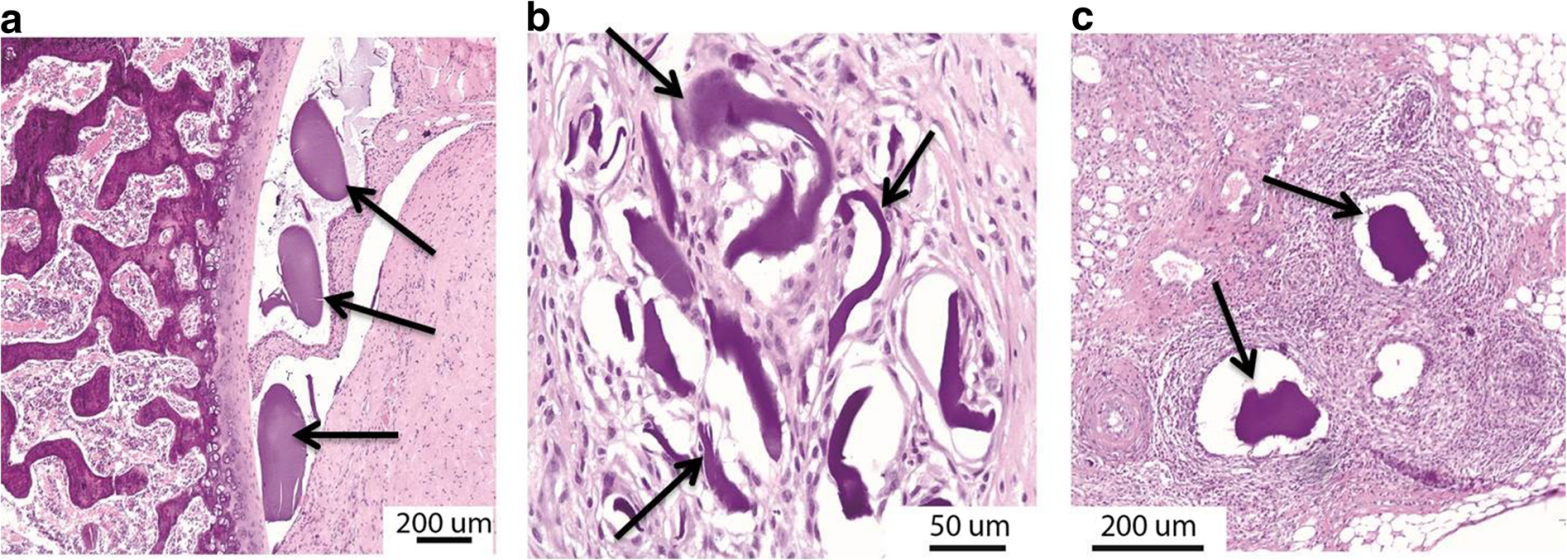
Empty alginate microbeads in healthy rat knees. HE staining one week after injection. (A+B)synovial thickening, encapsulation of the alginate beads. (C) hypercellularity in the synovium. Arrow indicate alginate. The shape of the beads may have changed due to the processing for histological analysis

As an indicator of inflammation, we performed thickness measurements of the synovial membrane at the para-patellar recesses at 4 and 8 weeks after start of treatment (Fig. 6g, h). No significant differences between groups were found at week 4 (*p* = 0.198) The hMSC-alginate group showed a trend toward a thicker membrane at week 8 (*p* = 0.058) and more infiltration of inflammatory cells next to encapsulation of alginate remnants (black arrows in Fig. 6i, j) compared with the saline control and free hMSC group. To examine if alginate would induce inflammation in the joint, we injected empty alginate beads intra-articularly in 2 additional healthy rat knees. One week after injection, synovial inflammation was seen on histology, characterized by synovial hypercellularity and encapsulation of the alginate beads, indicating a mild foreign body reaction against the alginate (Fig. 7).

## 4. Discussion

MSC have previously been described to have a beneficial effect in regenerative medicine, both in pre-clinical and some initial clinical studies, although evidence for long-term engraftment is low (van Buul et al. 2014; Diekman et al. 2013; Mak et al. 2016; Prockop 2009; von Bahr et al. 2012). This led Prockop et al. and von Bahr et al. to postulate the “hit-and-run” mechanism (Prockop 2009; von Bahr et al. 2012) which proposes the cells to only have a short interaction with the micro-environment. The design of the current study is based on the idea that the therapeutic efficacy of MSC could be enhanced by prolonging the local presence of MSC and their secreted factors at the desired location. To achieve this purpose, we encapsulated MSC in alginate and demonstrate that the cells remained viable in this carrier, and are protected against the allogeneic immune system and retained immunomodulatory capacity when stimulated by external cytokines or immune cells. Moreover, we demonstrate retention of construct integrity in vivo over time by longitudinal MRI. For this purpose, gadolinium was used to cross-link the alginate. By combining MRI with BLI of constructs that contained luciferase transfected cells, we showed that encapsulation of MSC is beneficial for in vivo cell survival and that it prolonged their local presence in a diseased and inflamed environment.

We used two types of alginate to encapsulate cells, both were clinical grade but differed in composition with respect to the ratio of guluronate and mannuronate. With both alginate types, MSC retained their immunomodulatory capacity in vitro*.* The results are similar to our previous study where we used a different type of alginate that had a low viscosity and less well-defined composition (Leijs et al. 2017). As there is a great variability in the ratio of mannuronate and guluronate between different types of alginate that are (commercially) available, our work in which we used high-quality GLP produced High G and High M alginates demonstrates that a wide range of alginates might be suitable for encapsulation. Different alginates have different viscosities which can greatly influence the mechanical properties of the construct and thus its integrity and the infiltration of cells. After subcutaneous implantation in immune competent rats, constructs of High G and High M alginates remained intact with a thin capsule formed around the construct. There was no infiltration of immune cells in the alginate. We took the alginate-encapsulated MSC a step further by evaluating them in a diseased situation: in our case, in rat knees after induction of osteoarthritis. To provide an injectable therapy, we optimized a protocol using a machine for encapsulation that enabled reproducible generation of a homogeneous population of MSC-alginate microbeads, with an average diameter below 300 μm. The size of these constructs contributes to easy clinical application since they are small enough to pass through a 23G needle that can be used for most clinical applications.

The use of gadolinium, with its contrast properties in MR imaging (Lux and Sherry 2018), made it possible to monitor localization and integrity of the alginate constructs over time. Gadolinium was incorporated in the guluronate or mannurate molecules upon polymerization, and loss of gadolinium signal was attributed to loss of construct integrity. Quantification with MRI of the Gd-labelled beads indicated an initial loss of some beads with subsequent retained visible presence of approximately half of the alginate beads up to the end of the experiments at 8 weeks post-injection. Although we cannot exclude that the loss of Gadolinium signal is caused partly by diffusion of gadolinium out of the bead, under in vitro conditions leakage of gadolinium out of the alginate beads was not seen at all during a 3-week follow-up period (data not shown). Therefore, we assume that lessening of the number of visible beads is due to disintegration of the beads with concomitant release and loss of hMSC. The latter is confirmed by the BLI data that showed a matching decrease in cell signal over time. A substantial part of the cells, however, remained present until the end of the study. Possibly, some beads are lost due to mechanical forces in the joint during movement of the animal. We speculate that this problem might be less in a larger joint where the beads have more space to be distributed to a relatively sheltered position, such as in the suprapatellar pouch, where high loading that occurs between cartilage surfaces can be avoided. The unique option to follow bead integrity on MRI, while having the anatomy of the joint visible in the same image, provides a safe and helpful tool to follow alginate constructs, also in a clinical setting in human, equine, or canine patients. The method might be useful for in vivo tracking of other materials that polymerize with divalent cations such as fibrin.

Besides bead and cell tracking to demonstrate prolonged cell presence, we tested therapeutic efficacy of the encapsulated MSC in a rat model for OA. Although we have previously shown MSC retain osteogenic and adipogenic differentiation capacity after 30 days of alginate encapsulation (Leijs et al. 2017), we hypothesize that therapeutic effect of MSCs is mainly by secretion of factors. In previous work, we have shown that multiple intra-articular injections of MSC secretome can inhibit pain and have a protective effect on cartilage damage in a mouse OA model (Khatab et al. 2018b). This confirms that MSC-based treatments can exert their effects in vivo by their secretome and do not rely solely on cell–cell contact or their differentiation capacity. In this study, we quantified the stimulation-induced IL-6 secretion and IDO activity from the encapsulated hMSC. This is, however, only a small fraction of the biologically active factors that are secreted by MSC, either soluble or in extracellular vesicles. It is, therefore, important to test the functionality of the secreted factors, which we did by demonstrating that these encapsulated hMSC significantly inhibited proliferation of stimulated CD4+ and CD8+ T lymphocytes in a dose-dependent manner.

Preferably, a continuous interaction and feedback loop between the diseased tissue and the exogenous MSC is created, in order to produce cytokines and growth factors at the right time and in the right concentration. Based on the longer presence, we choose to inject 1 × 10^5^ cells per joint. This number is ten times lower than what we injected previously in the same rat OA model (van Buul et al. 2014). Possibly as a consequence of that, in our study a therapeutic effect of freely injected human MSC was not detectable. The encapsulated MSC, however, did not do better than the freely injected MSC. This absence of improved therapeutic effect by encapsulation could be due to an insufficient number of cells. Maybe, initially a larger cell number is needed to reduce the inflammation. The small size of the rat joint, however, did not allow injection of more beads. Because preliminary experiments had indicated the density of 4 million cells/mL to be a good balance between concentration of secreted factors and stability of the gel construct, we have not considered using higher cell numbers per bead. Furthermore, we have chosen to use human MSC for this study to increase the clinical translatability of a human allogeneic MSC-alginate construct. A disadvantage of the use of xenogeneic MSC in this setup could be that some important factors and cytokines might not be interspecies conserved. This can cause in vivo miscommunication between xenogeneic MSC and the diseased environment. Since we and others have seen anti-inflammatory effects of xenogeneic MSC secretome alone, we can conclude that the secreted factors of xenogeneic MSC are capable of at least achieving anti-inflammatory and chondroprotective effects in OA (Khatab et al. 2018a). Nevertheless, it is still possible that the use of xenogeneic MSC depreciates the full potential of MSC therapy, an issue that could be tackled by using allogenic MSC. The use of xenogeneic MSC could also explain the discrepancy between our work and the recently published work of Choi et al., showing promising results using allogenic encapsulated MSC in a rabbit OA model, although in that study no cell or construct tracking was performed (Choi et al. 2018).

Although the use of alginate encapsulation is promising in the field of regenerative medicine, it might bring safety and regulatory issues. Although the fibrous capsule formed around the alginate implants when implanted subcutaneously was very thin and the constructs remained completely intact, upon injection in the joint, we noticed a trend to synovial thickening and the alginate beads were encapsulated in the synovial membrane. This reaction, even though it was not a strong foreign body response, might have dampened the anti-inflammatory effect of MSC and in extension its effect on pain. Since this reaction seemed less strong after subcutaneous implantation of MSC-alginate or empty alginate constructs, it might be caused by mechanical damage to the constructs or the presence of local inflammation in the osteoarthritic joint. If the alginate is compromised and starts to slowly release the xenogeneic hMSC, an adaptive immune response can be initiated, further reducing the therapeutic potential. Although immune privileged, MSC do maintain a degree of immunogenicity (Schu et al. 2012). This foreign body reaction leads possibly to a slow release of xenogenic MSC out of the alginate, possibly causing a chronic local inflammation. Thus, to limit this reaction, two factors play an important role: the biomaterial (the alginate) and the MSC. Focusing on the biomaterial, it is possible that a different type of alginate could be more resistant to damage in the osteoarthritic joint. This would prevent the release of xenogeneic hMSC, thus the adaptive immune response, and decrease the fibrous tissue formation as seen in our experiments. Another way to decrease this reaction is to use autologous MSC: this would further inhibit the graft vs. host disease. Of course, extensive in vitro and in vivo experiments are needed to investigate these hypotheses.

In conclusion, we have provided a method to produce a homogenous gadolinium-labelled cell-alginate construct combined with imaging techniques that are suitable for minimal invasive longitudinal follow-up studies in patients. We showed that non-autologous MSC can survive longer and remain metabolically active in vivo up to at least 8 weeks when encapsulated in alginate. The possibility to retain non-autologous cells and the production of standardized small beads greatly increased the feasibility of producing cell-alginate microcapsules in a standardized safe way and, on a large scale, giving it the potential of an “off-the-shelf” biological therapeutic option. These are both important additional steps toward clinical applicability. Unfortunately, the overall treatment effect on pain, synovial inflammation, and cartilage quality in this study could not be confirmed in our in vivo OA model, possibly due to specific local tissue responses to the alginate beads or a suboptimal cell number. Our results encourage further development of this strategy to provide an injectable therapy by cell encapsulation that greatly prolongs the interplay between the therapeutic cells and their diseased target tissues, taking into account specific local and disease requirements.
